# Carnosine and Beta-Alanine Supplementation in Human Medicine: Narrative Review and Critical Assessment

**DOI:** 10.3390/nu15071770

**Published:** 2023-04-05

**Authors:** Ondrej Cesak, Jitka Vostalova, Ales Vidlar, Petra Bastlova, Vladimir Student

**Affiliations:** 1 Department of Urology, University Hospital Olomouc, 775 20 Olomouc, Czech Republic; ondrej.cesak@fnol.cz (O.C.);; 2 Faculty of Medicine and Dentistry, Palacky University, 775 15 Olomouc, Czech Republic; 3 Department of Medical Chemistry and Biochemistry, Faculty of Medicine and Dentistry, Palacky University, 775 15 Olomouc, Czech Republic; 4 Department of Rehabilitaion, University Hospital Olomouc, 775 20 Olomouc, Czech Republic

**Keywords:** carnosine, beta-alanine, zinc-carnosine, supplementation, human diseases, health benefits

## Abstract

The dipeptide carnosine is a physiologically important molecule in the human body, commonly found in skeletal muscle and brain tissue. Beta-alanine is a limiting precursor of carnosine and is among the most used sports supplements for improving athletic performance. However, carnosine, its metabolite *N*-acetylcarnosine, and the synthetic derivative zinc-L-carnosine have recently been gaining popularity as supplements in human medicine. These molecules have a wide range of effects—principally with anti-inflammatory, antioxidant, antiglycation, anticarbonylation, calcium-regulatory, immunomodulatory and chelating properties. This review discusses results from recent studies focusing on the impact of this supplementation in several areas of human medicine. We queried PubMed, Web of Science, the National Library of Medicine and the Cochrane Library, employing a search strategy using database-specific keywords. Evidence showed that the supplementation had a beneficial impact in the prevention of sarcopenia, the preservation of cognitive abilities and the improvement of neurodegenerative disorders. Furthermore, the improvement of diabetes mellitus parameters and symptoms of oral mucositis was seen, as well as the regression of esophagitis and taste disorders after chemotherapy, the protection of the gastrointestinal mucosa and the support of *Helicobacter pylori* eradication treatment. However, in the areas of senile cataracts, cardiovascular disease, schizophrenia and autistic disorders, the results are inconclusive.

## 1. Introduction

**Figure 1:**
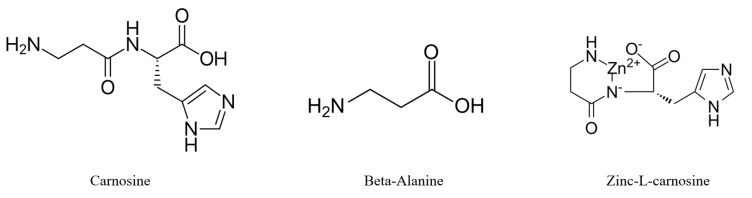
Chemical structure of carnosine, zinc-L-carnosine and beta-alanine.

Carnosine is a dipeptide composed of the non-proteogenic amino acid beta-alanine and the essential amino acid L-histidine (Figure 1). Carnosine is found in the human body not only in relatively high millimolar concentrations in excitable tissues (skeletal muscle and brain [1]) but also in smaller amounts in other tissues (gastrointestinal tract, kidney, liver, adipose tissue and heart) [2]. The bioavailability of beta-alanine limits the synthesis and amount of carnosine [3].

**Figure 2:**
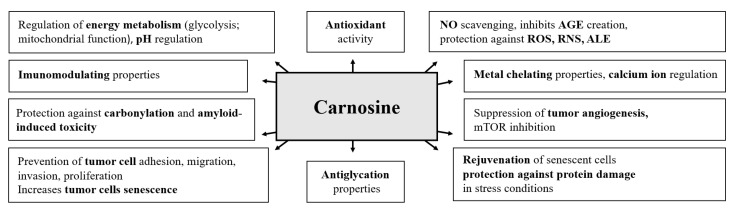
Biological activities of carnosine, simplified. ALE: advanced lipoxidation end products; AGE: advanced glycation end products; mTOR: mammalian target of rapamycin; NO: nitric oxide; RNS: reactive nitrogen species; ROS: reactive oxygen species. Additional information on carnosine’s molecular functions can be found in Supplementary Table S1.

Several biological effects have been described for carnosine and its precursor beta-alanine. At the cellular level, carnosine reacts with reactive oxygen species (ROS) and reactive nitrogen species (RNS), and oxidative damage products of biomolecules [4]. In this way, it protects other biomolecules from modification and damage [5]. Carnosine reduces not only the level of reactive carbonyls (RC) but also the formation of advanced glycation end products (AGEs) and advanced lipid peroxidation end products (ALE) [6,7]. Carnosine also has the ability to scavenge nitric oxide (NO) [8] and is involved in the chelation of transition metals [9]. Further, carnosine affects the regulation of calcium levels in muscle cells [10]. Carnosine can directly react with protons and, thus, participates in maintaining pH balance and reducing muscle fatigue caused by intense muscle activity [11]. Furthermore, carnosine is associated with the ability to modulate the endogenous antioxidant system by activating the signaling pathway controlled by the transcription factor Nrf2, which is involved in the removal and detoxification of oxidative modification products of biomolecules [11]. Carnosine also affects telomerase activity and slows down cell senescence [12], affects the resistance of proteins to heat or chemical stress [13] and has immunomodulatory effects [14]. It is also significantly involved in the regulation of intracellular metabolism, inhibiting glycolysis and increasing mitochondrial activity (the production of adenosine triphosphate, ATP) [15]. Finally, in vitro studies have revealed that carnosine has antineoplastic effects and affects the metabolism and senescence of tumor cells [15,16,17,18,19,20,21,22]. Figure 2 summarizes the published functions of carnosine.

Beta-alanine is one of the most used sports supplements worldwide [23], as it improves muscle performance in active athletes, specifically by increasing the concentration of carnosine in the muscles [24]. It is recommended for this purpose by the International Society of Sports Nutrition (ISSN) [25]. Besides carnosine and beta-alanine, *N*-acetylcarnosine (NAC) and the synthetically prepared derivative chelate compound zinc-L-carnosine (ZnC), also called Polaprezinc^®^ (containing 23% zinc and 77% L-carnosine), have biologically significant effects on the human body [26]. For a long time, the supplementation of carnosine and beta-alanine has been associated only with active athletes, but is now gaining popularity among persons with chronic diseases.

Current publications present recent data from a critical perspective, specifically from clinical trials in human medicine. This narrative review aims to increase knowledge of the clinical benefits of the supplementation with a focus on the primary and secondary prevention of diabetes mellitus; the prevention of sarcopenia; the improvement of cognitive abilities and neurodegenerative disorders; the treatment of schizophrenia, autism spectrum disorders and senile cataracts; and the improvement of prognoses of cardiovascular diseases, oral mucositis, taste disorders and other gastrointestinal diseases.

## 2. Search Strategy and Methodology

A narrative review was conducted between December 2021 and February 2023. Peer-reviewed journal articles were located from inception up to January 2023. The search strategy was aimed at evaluating clinical studies on the role of carnosine and beta-alanine supplementation in human clinical studies. Scientific articles were searched for using PubMed, Web of Science, the National Library of Medicine and the Cochrane Library databases. A search strategy using database-specific keywords was employed. The search terms used included “carnosine”, “beta-alanine”, “zinc-carnosine”, “polaprezinc”, “supplementation” combined with “cataract” and/or “neurodegenerative” and/or “mucositis” and/or “diabetes mellitus” and/or “sarcopenia” and/or “gastroenterology” and/or “psychiatry” and/or “cardiovascular system” and/or “schizophrenia” and/or “ADHD”. Only papers with English translations were reviewed. The search retrieved approximately 800 articles and 118 were used in the present review.

## 3. Synthesis and Degradation

Carnosine is synthesized in the human body from beta-alanine and histidine [27]. The synthesis is catalyzed by ATP-dependent carnosine synthetase and the rate of the intracellular synthesis is greatly limited by the level of beta-alanine [28]. Beta-alanine itself is synthesized in the liver (during the catabolism of polyamines, pyrimidines and coenzyme A; the bacterial catabolism of aspartate; and the transamination of malonic acid semi aldehyde [27]) and then transported to muscle and brain cells, where it is utilized for carnosine synthesis [2]. It can be estimated that a 60 kg woman and a 70 kg man synthesize 427 and 606 mg of carnosine, respectively, per day [29].

The intracellular concentration of carnosine depends on the hydrolytic activity of carnosinases (CN 1 or CN 2) and the synthetic activity of carnosine synthetase, and is greatly limited by the dietary uptake of beta-alanine and essential amino acid histidine [10].

Dietary carnosine and beta-alanine are absorbed in the human small intestine on the apical side by specific transporters: carnosine by a peptide transporter, beta-alanine by a specific transporter for beta-amino acids and taurine, and histidine by a sodium dependent neutral amino acid transporter [2]. In enterocytes, carnosine is hydrolyzed by CN 2 [2]. Carnosine and histidine cross the basolateral membrane of enterocytes to the blood by a proton coupled transporter, and beta-alanine by a specific transporter for beta-amino acids [2]. Nearly all ingested carnosine enters the portal circulation [30]. In the blood, carnosine is hydrolyzed by CN1 and the half-life of carnosine in the human serum is under 5 min [31]. Histidine and beta-alanine cross the membrane of target cells in the extraintestinal tissues, mainly, muscle, the liver and the brain, by specific transporters [2], where they are used for the synthesis of carnosine.

The carnosine molecule is enzymatically hydrolyzed in the human body by CN1 and CN2 [31], which act as the regulators of carnosine level [32].

In the central nervous system (CNS) [33], carnosine is hydrolyzed, and the resulting histidine is enzymatically converted by histidine decarboxylase in specific parts of the brain to the neurotransmitter histamine [34].

**Figure 3:**
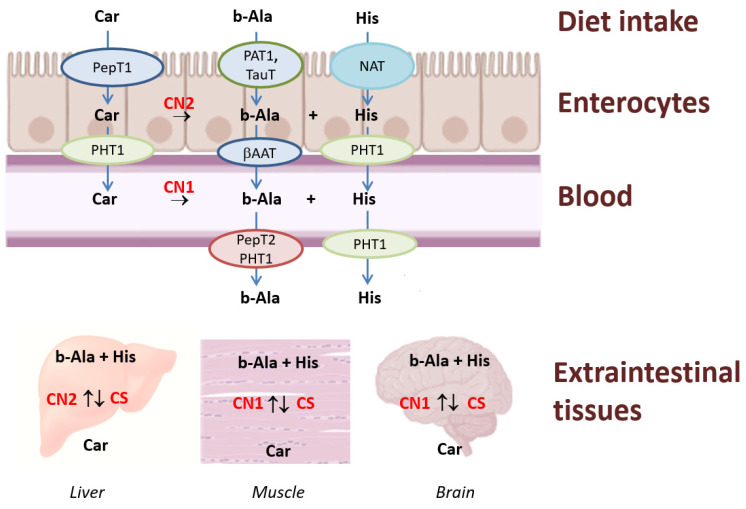
Metabolism of carnosine and beta-alanine. Carnosine (Car) enters the enterocyte by a peptide transporter (PepT1); beta-alanine (b-Ala) enters by a specific transporter for beta-amino acids, a proton-assisted amino acid transporter (PAT1) and a taurine transporter (TauT); and histidine (His) enters by a sodium-dependent neutral amino acid transporter (NAT). Proton-coupled peptide-histidine transporter 2 (PepT2) carnosine transporting is expressed in the membrane on majority extraintestinal tissue. b-Ala is transported from the circulation by beta-amino acid specific transporter (ßAAT) and His by PHT1. In the extraintestinal tissues, carnosine is cleaved by carnosinases CN1 or CN2 into b-Ala and His, respectively, and formed from these two amino acids through carnosine synthetase (CS).

The metabolism of carnosine and beta-alanine, along with their synthesis and degradation cycle, is summarized in Figure 3.

## 4. Supplementation and Food Sources

Increasing the dietary intake of carnosine enhances its concentrations in, mostly, skeletal muscle, the brain and the heart [10]. An important source of carnosine is provided by foods such as chicken meat [35], fish and shrimp, as well as asparagus, green peas and white mushrooms [36]. Interestingly, it is suggested that some components in beef inhibit serum CN1 [10]. A daily dietary intake of 30 g of dried beef should be able to completely supply daily carnosine requirement of a 70 kg adult to ameliorate human nutrition and health [2]. Even though its limiting precursor beta-alanine is produced endogenously in the liver, its main source is from a person’s diet. Humans acquire beta-alanine through the consumption of foods with large beta-alanine content, such as poultry, beef and fish [25]. The administration of beta-alanine seems to be more convenient and effective than carnosine, because the bioavailability of carnosine is reduced by CN activity [31]; however, beta-alanine is reused for carnosine synthesis by skeletal muscle, the heart, and the olfactory bulb of the brain.

In a meta-analysis of the adverse effects and risks of oral beta-alanine supplementation, no adverse effects on human health were observed (at doses from 4 to 6 g per day) [23]. The only adverse effect was paresthesia and a small increase in alanine aminotransferase activity was described (the increase was within reference ranges) [23]. There is no risk of overdose when carnosine is administered because carnosine is cleaved by CN1 into amino acids, which are then involved in metabolism [10].

## 5. Carnosine, Beta-Alanine and Diabetes Mellitus

The formation of AGEs and RC is one of the causes of diabetic complications. In patients with type 2 diabetes mellitus (T2DM), RC levels correlate with insulin resistance [37]. Carnosine can interact with these reactive molecules and, thus, prevent the development of adverse complications in patients with diabetes mellitus (DM) [38].

The potential of carnosine supplementation in the prevention of T2DM was described in a clinical trial by de Courten et al. [39]. The administration of carnosine (2 g/day for 12 weeks) led to a reduction in fasting insulin levels and a reduction in insulin resistance [39]. Furthermore, carnosine supplementation in a randomized placebo-controlled trial has shown a nephroprotective effect (the reduction in urinary transforming growth factor-beta) in 40 patients with diabetic nephropathy [40]. The effect of supplementation was also suggested by a different randomized trial, in which it reduced glycaemia in 82 patients with T2DM; however, this study combined carnosine supplementation and 2 other supplements, so this effect cannot be attributed to carnosine alone [41]. The benefits of beta-alanine administration (4 g/day in 3 doses, over 28 days) in combination with increased physical activity in patients with T2DM led to a reduction in glycaemia and a simultaneous improvement in physical capacity [42]. Another placebo-controlled randomized trial described the effect of carnosine supplementation (500 mg twice daily, over 12 weeks) on complications of type 1 diabetes (T1DM) in children and adolescents. The dose was well tolerated by the subjects, and a decrease in parameters capturing oxidative stress, an increase in antioxidant capacity and an improvement in glycated hemoglobin (HbA1c) and renal metabolism (alpha-1 microglobulin) were observed [43]. After carnosine supplementation (2 × 500 mg/day, over 12 weeks) in T2DM patients, fasting glycaemia, HbA1c, serum triglyceride (TAG) and tumor necrosis factor-alpha (TNF-α) levels were lowered [44].

A meta-analysis showed that supplementation with carnosine and beta-alanine led to reductions in fasting glycaemia, a decrease in HbA1c levels and insulin resistance [45]. Another meta-analysis including only randomized trials confirmed the effect of carnosine supplementation on DM patients, specifically on their HbA1c and fasting glucose levels [46]. This is supported by the results of yet another meta-analysis on 30 clinical trials of the effect of carnosine and related dipeptides in obese subjects, which showed reductions in fasting glycaemia and HbA1c, as well as reductions in obesity (waist circumference) [47]. Available trials on the effectiveness of supplementation are summarized in Table 1.

Findings from clinical trials show that supplementation with carnosine (ranging from 500 mg to 2 g per day) and beta-alanine (4 g per day) is important in preventing and slowing the progression of T2DM. At the same time, it reduces complications associated with obesity, T1DM and T2DM, including oxidative stress, and improves carbohydrate metabolism [45,46,47].

## 6. Carnosine, Beta-Alanine, and Neurological Diseases

In addition to muscle tissue, carnosine is also found in the CNS. Due to its properties, carnosine participates in the maintenance of homeostasis in the CNS, acting as a neuroprotective agent [48]. Through the mechanism of neuroprotection by reactions with ROS, RNS, AGEs and RC, it has the potential to promote cognitive maintenance and prevent the development of neurodegenerative diseases. Even the precursor of carnosine, beta-alanine, affects many neurocirculatory processes through competitive inhibition of taurine [49]. The activity of CN1 increases with age, leading to a lower accumulation of carnosine in muscle, the brain and other tissues [50]. This is also related to the clinical entity of dementia (specifically in the aging population), which is accompanied by increased production of ROS, RNS and AGEs and inflammation [51].

The increasing prevalence of neurological and neurodegenerative diseases is a social issue. These diseases are often associated with disability and their treatment is still inadequate. The potential of carnosine or beta-alanine supplementation in clinical trials was extensively described in the review by Schön et al., in which the authors describe promising results in the field of neurology, for diseases such as Alzheimer’s disease (AD), Parkinson’s disease (PD), and multiple sclerosis [52]. This section of the review presents the results of selected clinical trials focusing on the CNS, and available studies are summarized in Table 2.

The investigation of improving cognition and slowing the progression of neurodegenerative diseases was conducted in an interventional, double-blind, placebo-controlled trial by Rokicki et al. [53]. The results showed that supplementation with carnosine in combination with anserine (500 mg in a 1:3 ratio, for 3 months) had a positive cognitive effect in elderly people (40–78 years) [53]. Similarly, another double-blind trial in an elderly population (56 subjects aged 65+) combining carnosine with anserine (2.5 g of anserine and carnosine in a 2:1 ratio, for 13 weeks) revealed improvements in cognitive function and physical capacity [54]. Another study that investigated supplementation of carnosine with anserine (1 g of carnosine and anserine in a 3:1 ratio for 3 months) in elderly patients resulted in a significant improvement in verbal memory and correlated with the suppression of the inflammatory chemokine CCL24, which may be responsible for these cognitive symptoms [55]. However, none of the previous three trials could completely attribute the results to carnosine, given the ratio of active ingredients.

Hisatsune et al. observed a decrease in blood levels of pro-inflammatory cytokines and an increase in cerebral blood flow after carnosine supplementation [56]. The effect of carnosine was studied in a double-blind randomized trial on Gulf War veterans who suffered from complex cognitive and multisystemic symptoms (Gulf War Illness, GWI, or Chronic Multisymptom Illness). The etiology of GWI is thought to be associated with increased ROS production. Study participants were given increasing doses of carnosine (0.5–1 g daily at 4-week intervals for 12 weeks), which significantly improved their Wechsler Adult Intelligence Test scores [57]. Solis et al. did not confirm an effect on cognitive function after beta-alanine supplementation (6.4 g/day for 28 days), but this trial was limited by the small number of participants [58].

The beneficial properties of carnosine and its components (beta-alanine and histidine) have been described in the additional literature [53,56,57]. A meta-analysis of randomized trials confirmed the effects of carnosine supplementation on the Wechsler memory scale-revised logical memory delayed recall (WMS-LM2), specifically in doses of around 300 mg of carnosine per day [46].

### Neurodegenerative Diseases

Globally, neurodegenerative diseases represent a significant burden on the healthcare system. Three of the major neurodegenerative diseases are AD, PD and amyotrophic lateral sclerosis (ALS). The prevalence and incidence of these diseases increase dramatically with age, and, therefore, the number of cases is expected to increase as life expectancy continues to rise [62].

Patients with probable AD (pAD) showed changes in plasma amino acids, including a reduction in carnosine levels, which may be related to reduced antioxidant capacity in AD patients [63].

After supplementation with anserine and carnosine (54 subjects; 750 and 250 mg/day for 12 weeks), no significant effect on cognitive function was observed in patients with a mild form of cognitive dysfunction; however, in a follow-up analysis, it was found that there was an improvement in cognitive function in participants who are Apo E4 carriers (Apo E4 functions as a risk factor for AD development). For these patients, supplementation had a protective function against cognitive deterioration in patients with mild cognitive impairment [59]. Only one randomized placebo-controlled trial investigated carnosine supplementation (1.5 g/day for 1 month) in 36 patients with PD, according to the basic treatment protocol. Carnosine led to improvements in neurological symptoms associated with improved antioxidant capacity. The administration of carnosine together with the basic therapy not only improved the clinical parameters but also improved the antioxidant status of the PD patients [60].

In another clinical trial, the effect of a mixture of antioxidants and vitamins, including carnosine (100 mg/day), on AD patients treated with donepezil was investigated. After a six-month treatment with this mixture, a reduction in oxidative stress parameters and an improvement in mini-mental state examination, version 2 (MMSE-2) scores were observed in the donepezil-treated group when compared to the placebo [61].

Slowing the progression and improving the symptoms of certain neurodegenerative diseases, specifically AD and PD, has been confirmed sporadically in clinical trials in specific patient groups [59,60,61,64]. A meta-analysis of randomized trials confirmed the positive effect of carnosine supplementation (in doses of up to 1.5 g per day) in the Alzheimer’s Disease Assessment Scale and the Beck’s Depression Inventory [46]. Unfortunately, the number of clinical trials is very small. Nevertheless, we suggest that preventive long-term supplementation with these substances in the aging population could slow down and delay the onset of degenerative changes in the CNS. High-quality clinical studies are needed to confirm this theory.

## 7. Carnosine, Beta-Alanine, and Psychiatric Diseases

The increasing prevalence of psychiatric illness is considered a major social problem. Recently, there has been an increase in the number of cases of psychiatric illnesses and associated rising demands on the healthcare system worldwide. For example, the negative symptoms of schizophrenia, with a prevalence of about 25%, are a challenging aspect of this disease, precisely because of the inconsistent efficacy of current antipsychotic medication [65]. Several mechanisms are thought to be responsible for the negative symptoms, such as, for example, the effect of the disease on glutaminergic synapses [66]. Carnosine and its effect on these synapses could lead to the clinical effect of reducing these symptoms of schizophrenia. The potential of supplementation in clinical trials has been extensively described in a review by Schön et al., in which the authors present promising results of carnosine or beta-alanine supplementation in clinical trials concerning psychiatric disorders, autism spectrum disorders and many others [52].

An 8-month randomized double-blind placebo-controlled trial of 60 patients with chronic schizophrenia treated with risperidone focused on studying the effects of carnosine (2 g/day). The administration of carnosine along with therapy resulted in a reduction in negative symptoms of schizophrenia without an increase in side effects [67]. This was confirmed in a randomized trial of 75 patients with chronic schizophrenia, in which carnosine supplementation (2 g/day for 3 months) improved executive functions [68].

In addition, the depletion of beta-alanine and histidine may not occur in active disease exclusively. Increase in the levels of these amino acids might show potential to monitor treatment effect [52]. In a study of patients with depressive disorders treated with selective serotonin reuptake inhibitors (SSRIs), plasma beta-alanine levels were decreased compared to healthy controls [69]. This has also been indicated in people suffering from depression. The use of the antidepressant quetiapine led to a decrease in carnosine levels. If the medication was not administered, there was a gradual increase in serum carnosine levels over the course of the depression (over 40 months). The authors assume that this is a defensive reaction of the organism to this condition [70].

Isolated published studies suggest a benefit of carnosine supplementation on schizophrenia symptomatology [67,68], but further high-quality studies are needed to confirm the benefit in practice. The compounds’ ability to monitor treatment effect is only theoretically suggested, currently, without robust data to support it.

### Autism Spectrum Disorders in Children

Autism spectrum disorders (ASD) are complex disorders accompanied by impairments in certain brain functions and represent a global healthcare issue. The etiology of ASD is multifactorial [71]. Studies show that imbalances in transit metals (increased levels of lead, cadmium, mercury and nickel and decreased levels of zinc) can disrupt mitochondrial function and antioxidant capacity; induce synapse dysfunction; impair myelination; and affect neurogenesis and neural differentiation, synapses, myelination and neuroinflammation development [72,73]. Carnosine could reduce oxidative stress in ASD sufferers due to its antioxidative properties and ability to chelate transit metals.

Most clinical trials evaluating levels of carnosine and similar peptides in neurological and neurodevelopmental disorders have been conducted in children with ASD [74]. Reduced levels of carnosine, including histidine and beta-alanine, have also been demonstrated in children and adolescents with ASD [75,76], suggesting the potential use of carnosine in the treatment of autistic patients.

Carnosine supplementation (800 mg/day for 8 weeks) resulted in improved communication skills and behavior in autistic children. The authors also reported improvements in receptive speech and social attention, a reduction in apraxia and an overall improvement in brain function [77]. Mehrazad-Saber et al. observed a reduction in the frequency of sleep disturbances in children with ASD with carnosine supplementation (500 mg daily for 2 months), but the effect on the course of the disease was not confirmed in the trial [78]. In a double-blind randomized trial of 70 children with ASD, carnosine supplementation (800 mg daily for 10 weeks) resulted in a reduction in hyperactivity and non-compliance [79], although these results were not confirmed in another randomized placebo-controlled trial on 63 participants [74]. The effectiveness and safety of carnosine has also been studied in children with ADHD (Attention-Deficit/Hyperactivity Disorder). In a randomized double-blind placebo-controlled trial, either carnosine or a placebo was administered in addition to methylphenidate (0.5–1.5 mg/kg/day). Treatment with carnosine (2 g/day for 8 weeks) and risperidone showed good tolerability and significant beneficial effects on negative symptoms in patients with stable disease [80].

Studies with carnosine have pointed to its potential use in improving sleep in children and adolescents with ASD; however, Esposito et al., in their review, stress the need for further research to confirm the efficacy of this molecule for the treatment of sleep disorders in patients with ASD [81]. Although it appears from the results of individual trials that the administration of carnosine, or its components beta-alanine and histidine, could be beneficial for children with ASD [77,78,79,80], carnosine was also included in a meta-analysis on the use of drugs and dietary supplements in ASD, and the available data were considered insufficient and, therefore, carnosine cannot be therapeutically recommended [82]. Based on the results of a clinical trials meta-analysis on the effect of carnosine supplementation (up to 2 g per day) on children with ASD (only 5 eligible studies with 215 participants were selected), it is not possible to recommend carnosine supplementation for children with ASD [74].

Further high-quality clinical trials with a larger number of patients will be needed to verify the effect of these agents on children’s health and possibly recommend them as an add-on to conventional therapy. Available trials are summarized in Table 3.

## 8. Carnosine, Beta-Alanine and Cataract

Cataracts are a global healthcare concern in both developed and developing countries. The main cause of cataracts is aging, but it is also related to other exogenous factors such as UV radiation and trauma, and endogenous factors such as DM or hereditary predisposition [84]. The potential of carnosine in slowing the process of degenerative changes in vision and in the treatment of senile cataracts itself is summarized in the article by Wang et al. [85]. Although cataract surgery is effective and safe, using an antioxidant in the form of topical carnosine eye drops can provide patients with another treatment option. Carnosine might have the potential, through its antioxidant and antiglycation properties [7], to delay visual impairment with aging, effectively preventing and treating senile cataracts [85]. The beneficial effect of NAC on senile cataracts was found in 49 elderly patients (mean age 65 years) with variously advanced cataracts in a randomized, placebo-controlled, double-blind trial with eye drops of 1% aqueous NAC solution (2 drops/2 times daily for 6 and 24 months) [86]. NAC’s positive effect on eye health and its safety have also been described in other clinical trials from the same author [87,88].

Although the results of the abovementioned trials appear promising, an externally conducted analysis of randomized clinical trials (including only two randomized trials) concluded that there is no credible evidence at this time that NAC has preventive effects against, or that it slows the development of, cataracts. The primary reason for this decision was the lack of information on the methodology of the trials conducted [89]. Furthermore, well-designed clinical trials with a larger number of participants will be needed to confirm or refute the effect.

## 9. Carnosine, Beta-Alanine and Sarcopenia

Sarcopenia is a complex and multifactorial progressive muscle disorder that develops with age. In elderly patients, sarcopenia is associated with a greater likelihood of adverse events, including falls, fractures, physical disability and even mortality [90]. The deterioration of the human organism in old age may be associated with reduced tissue concentrations of carnosine, and, therefore, a lack of antioxidant capacities [91]. The activity of CN increases with age, leading to less accumulation of carnosine in muscle, the brain and other tissues [50]. It is believed that a diet rich in carnosine can slow the process of sarcopenia, aging and the development of age-related diseases [92,93]. Considering the confirmed functions of carnosine, increasing its levels in muscle could at least partially slow down the progression of sarcopenia.

In a double-blind, placebo-controlled trial of 18 subjects (60–80 years), supplementation with the carnosine precursor beta-alanine (1.6 g twice daily for 12 weeks) resulted in an increase in muscle carnosine content and improved physical performance [94]. In a double-blind placebo-controlled trial, when 2 doses of beta-alanine (800 mg and 1200 mg for 12 weeks) were administered, a significant increase in work capacity was seen compared to the placebo group [95]. Similar conclusions were reached in a trial that found a positive effect of beta-alanine (2.4 g/day) on exercise performance and subsequent muscle recovery in elderly subjects (60.5 ± 8.6 years) [96].

Supplementation with beta-alanine (in doses up to 3.2 g per day) could potentially represent one possible strategy to increase muscle activity in old age and slow down the sarcopenia process. This dietary intervention represents one option that could, therefore, lead to increased muscle performance and delay the problems associated with sarcopenia, thereby improving the quality of life of the elderly [94,95,96]. Available trials are summarized in Table 4.

## 10. Carnosine, Beta-Alanine and Diseases of the Cardiovascular System

Cardiovascular disease (CVD) remains the leading cause of death globally. Endothelial dysfunction represents one of the earliest pathophysiological factors leading to the development of CVD [97]. Carnosine may also be useful in the treatment and prevention of CVD [97]. Both in vitro and experimental studies have documented the benefits of carnosine supplementation in reducing the risk of atherosclerosis [98] and have described anti-ischemic effects [99].

One of the few studies in human medicine to address carnosine’s effect on CVD, a randomized placebo-controlled clinical trial, found that adding carnosine (500 mg/day for 6 months) to established treatments for chronic heart failure improved cardiopulmonary stress tests, 6-min walk test scores and quality of life [100]. In a randomized placebo-controlled trial of 50 patients after acute myocardial infarction and percutaneous coronary intervention, the anti-inflammatory effect of ZnC (75 mg twice daily for 9 months) was confirmed along with an improvement in cardiac function compared to the placebo group [101].

In some experimental studies, mostly performed in vitro, the added value of carnosine supplementation is suggested [98,99]. The results of very few human clinical trials are available [100,101] and the effect was not confirmed in the published meta-analysis [46]. High-quality clinical trials confirming these mechanisms are still lacking.

## 11. Zinc-Carnosine and Oral Mucositis, Loss of Taste and Gastrointestinal Tract

Zinc is a biogenic element that is essential for the proper function of a wide range of biomolecules. Zinc acts as a cofactor of a number of antioxidant enzymes and is essential for cell proliferation and cell repair [102]. ZnC has several biological effects, including maintenance, prevention and treatment of mucosal and epithelial tissue damage [26,103,104].

Loss of taste is a common side effect of chemoradiotherapy. Oral mucositis, oesophagitis and other gastrointestinal complications are common following radiotherapy or chemotherapy. Although these are not life-threatening conditions, they significantly reduce quality of life, and, therefore, it makes sense to investigate the possibilities of their prevention and treatment. Since Zn is involved in the healing processes of connective and epithelial tissue [105], the next part of the review is devoted to Zn.

Evidence supports the use of ZnC in the maintenance, prevention and treatment of mucosal and epithelial tissue damage, and in the treatment of oral mucositis and taste disorders in cancer patients undergoing radiotherapy and chemotherapy [104].

The efficacy of ZnC was confirmed in trials investigating taste receptors, in which ZnC supplementation (17 mg, 34 mg or 68 mg/day for 12 weeks) resulted in a faster return of taste [106]. Patients with taste dysfunction experienced faster symptom disappearance after ZnC supplementation (150 mg twice a day until symptom disappearance) [107]. A prospective trial on patients with radiation-induced oral mucositis confirmed the effect of an oral ZnC rinse, which led to a lower incidence of grade 3 oral mucositis, both by mucosal findings and subjective symptomatology [108]. Similar benefits were also described by Hayashi et al. [109,110]. In a trial of patients with hematological malignancies after chemoradiotherapy followed by hematopoietic stem cell transplantation, a benefit was also demonstrated. Patients rinsed their mouths with ZnC mouthwash containing sodium alginate (P-AG), which reduced the incidence of moderate to severe oral mucositis [109]. P-AG has also been shown to reduce the time to hospital discharge after radiotherapy in patients with head and neck cancer [111]. In a trial involving 10 patients, ZnC was found to reduce damage to the stomach and small intestine and contribute to gastrointestinal mucosal stabilization [112]. In one randomized study, ZnC oral rinse had a beneficial effect when it came to the use of analgesics [113]. Available trials on the effectiveness of ZnC supplementation are summarized in Table 5.

However, in another study, the effect of ZnC supplementation was not described [116]. ZnC supplementation reduced the incidence of esophagitis by more than two grades and delayed the onset of grade 2 esophagitis [114].

Supplementation of ZnC (4 × 18.75 mg/day) affected mucositis grade >2 in a trial involving 88 patients after high-dose chemotherapy before hematopoietic cell transplantation [118]. Supplementation also reduced diarrhea associated with irritable bowel syndrome [57], and reduced the incidence of radiation-induced stomatitis in a placebo-controlled trial [117].

ZnC as a supplement has been approved in the USA, Canada and Australia for use in the prevention and mitigation of post-radiation oral mucositis, loss of taste after chemotherapy and as a regulator of zinc release into tissue structures [120]. In Japan and South Korea, ZnC is prescribed for use in the treatment of surgical wounds after the removal of gastric ulcers and the eradication of *Helicobacter pylori* [115]. Its usability as an add-on therapy (omeprazole 20 mg, amoxicillin 1 g and clarithromycin 500 mg, each twice daily) for the eradication of *H. pylori* was confirmed in a randomized placebo-controlled prospective multicenter trial [115]. ZnC (150 mg/day) and proton pump inhibitor together showed non-inferiority to the standard treatment in the gastric ulcer healing rate [119]. ZnC is officially approved for this purpose in Japan [120]. A review by Hewlings et al. described the effect of ZnC on oral mucositis, taste disorders and the gastrointestinal system and presented more evidence in the area of promoting the mucosal integrity of the gastrointestinal tract [120].

ZnC has also been investigated by the European Food Safety Authority (EFSA), which mentioned limited bioavailability, since ZnC is insoluble in water at neutral pH, and also expressed reservations about the nature of the particles being absorbed [26]. ZnC is absorbed by the human body and effectively provides zinc to the tissues [26]. The molecule has also been reviewed for safety and use in humans by the US Food and Drug Administration (FDA) and was granted “new dietary ingredient” status in 2002 [121].

## 12. Conclusions

Data from clinical trials demonstrate the potential benefits of beta-alanine (4–6 g per day) and carnosine (up to 2 g per day) supplementation mainly in the prevention of sarcopenia, neuroprotection and cognitive preservation, neurodegenerative diseases and the improvement of diabetes mellitus parameters. The effectiveness has also been demonstrated for a synthetic derivative of zinc-L-carnosine (68–500 mg per day) to improve symptoms and clinical findings of oral mucositis, to treat and prevent taste disorders after chemotherapy, to regress symptoms of esophagitis, to protect the gastrointestinal mucosal layer and as an additive to the eradication treatment of *Helicobacter pylori*.

Conversely, in the areas of senile cataracts, cardiovascular disease, autistic disorders in children and schizophrenia in adults, the results to date are inconclusive and further clinical trials are needed to confirm a possible effect.

## Figures and Tables

**Table 1:** Carnosine, beta-alanine and diabetes mellitus.

Author (Year)	Study Design	Intervention	Number of Patients	Effect
de Courten et al. (2016) [39]	double-blind RCT	Carnosine orally (2 g in 2 doses/day) or placebo; 12 weeks	30	Preserved insulin sensitivity and insulin secretion, normalized glucose intolerance and reduced 2-h insulin levels after o-GTT in a subgroup of individuals with impaired glucose tolerance
Siriwattanasit et al. (2021) [40]	RCT	Carnosine (2 g/day) or placebo; 12 weeks	40	Nephroprotective effect of oral supplementation to decrease urinary TGF-β.
Karkabounas et al. (2018) [41]	double-blind RCT	Alpha-lipoic acid (7 mg/kg bodyweight), carnosine (6 mg/kg bodyweight), thiamine (1 mg/kg bodyweight) or placebo; 8 weeks	82	Supplementation effectively reduced glucose concentration in patients with T2DM.
Nealon et al. (2016) [42]	double-blind RCT	Beta-alanine (4 g split into 3 doses/day) or placebo; 28 days	12	Beta-alanine supplementation can increase exercise capacity in individuals with T2DM.
Elbarbary et al. (2018) [43]	double-blind RCT	Patients with diabetic nephropathy received supplemented carnosine (1 g/day) or placebo; 12 weeks	90	Oral supplementation with L-carnosine for 12 weeks resulted in a significant improvement of oxidative stress, glycemic control and renal function.
Houjeghani et al. (2018) [44]	double-blind RCT	Patients with T2DM received carnosine (500 mg, 2×/day, capsules) or placebo	54	Carnosine supplementation lowered fasting glucose, serum levels of triglycerides, AGEs and tumor necrosis factor-α without changing sRAGE, IL-6 or IL-1β levels in T2DM patients.

AGEs: advanced glycation end products; IL-6: interleukin 6; IL-1β: interleukin 1-beta; o-GTT: oral glucose tolerance test; RCT: randomized controlled trial; sRAGE: soluble receptor for advanced glycation end products; TGF-β: transforming growth factor-β; T2DM: type II diabetes mellitus.

**Table 2:** Carnosine, beta-alanine and neurological diseases.

Author (Year)	Study Design	Intervention	Number of Patients	Effect
Rokicki et al. (2015) [53]	double-blind RCT	Carnosine/anserine (1:3 ratio; 500 mg/day) or placebo; 3 months	31	Intervention group had better verbal episodic memory performance and decreased connectivity in the default mode network, the posterior cingulate cortex and the right frontal parietal network. A correlation between the extent of cognitive and neuroimaging changes was observed.
Szcześniak et al. (2014) [54]	RCT	Chicken meat extract containing anserine and carnosine (2:1 ratio; 1 g/day) or placebo; 13 weeks	51	Mean values of Short Test of Mental Status (STMS) scores increased in the intervention group (in the subscores of construction/copying, abstraction and recall), and promising effects on physical capacity.
Katakura et al. (2017) [55]	double-blind RCT	Anserine/carnosine (3:1 ratio; 1 g/day) or placebo; 3 months	60	Supplementation may preserve verbal episodic memory, probably owing to inflammatory chemokine CCL24 suppression in the blood.
Hisatsune et al. (2015) [56]	double-blind RCT	Anserine/carnosine (3:1 ratio; 1 g/day) or placebo; 3 months	39	MRI analysis showed a suppression in the age-related decline in brain blood flow in the posterior cingulate cortex area. Delayed recall verbal memory showed significant preservation in the intervention group.
Baraniuk et al. (2013) [57]	double-blind RCT	Carnosine (500, 1000 and 1500 mg/day, increasing at 4-week intervals) or placebo; 12 weeks	25	Supplementation may have beneficial cognitive effects. Fatigue, pain, hyperalgesia, activity and other outcomes were resistant to treatment.
Solis et al. (2015) [58]	double-blind RCT	Beta-alanine (6.4 g/day) supplementation or placebo; 28 days	19	Supplementation did not influence cognitive function before or after exercise in trained cyclists.
Masuoka et al. (2019) [59]	double-blind RCT	Anserine (750 mg/day) and carnosine (250 mg/day) or placebo; 12 weeks	54	Protective effects against cognitive decline in APOE4 (+) MCI subjects exist.
Boldyrev et al. (2008) [60]	two-arm, prospective	In addition to basic PD therapy, carnosine (1.5 g/day); 30 days	36	The combination of carnosine with basic therapy may be a useful way to increase efficiency of PD treatment.
Cornelli et al. (2010) [61]	two-arm, RCT	Carnosine (100 mg/day) with a mixture of antioxidants (beta-carotene, selenium, cysteine, ginko biloba and coenzyme Q10) and vitamins (B1, B2, B3, B6, B9, B12, C, D and E) on Alzheimer’s disease (AD) patients treated with donepezil; 6 months	52	A reduction in oxidative stress parameters and an improvement in mini-mental state examination, version 2 (MMSE-2) scores were observed.

AD: Alzheimer’s disease; MCI; Mild Cognitive Impairment; MMSE-2: mini-mental state examination, version 2; PD: Parkinson’s disease; RCT: randomized controlled trial; STMS: Short Test of Mental Status.

**Table 3:** Carnosine, beta-alanine and psychiatric diseases and their monitoring treatment effects.

Author (Year)	Study Design	Intervention	Number of Patients	Effect
Ghajar et al. (2018) [80]	double-blind RCT	Carnosine (2 g/day in two doses) or placebo; 8 weeks	60	Administration of carnosine along with therapy resulted in a reduction in negative symptoms of schizophrenia without an increase in side effects.
Chengappa et al. (2012) [68]	double-blind RCT	Carnosine (2 g/day) or placebo; 3 months	75	Intervention group performed significantly faster on non-reversal condition trials of the set-shifting test. The strategic target detection test displayed improved strategic efficiency and fewer perseverative errors.
Chez et al. (2002) [77]	double-blind RCT	Carnosine supplementation (800 mg/day) or placebo; 8 weeks	31	Improved communication skills and behavior in children with ASD. The authors also reported improvements in receptive speech and social attention, a reduction in apraxia and an overall improvement in brain function.
Mehrazad-Saber et al. (2018) [78]	double-blind RCT	Carnosine (500 mg/day) or placebo; 2 months	43	Carnosine supplementation did not change anthropometric indices and showed no effect on autism severity, whereas it significantly reduced sleep duration, parasomnias and total sleep disorders scores.
Hajizadeh-Zaker et al. (2018) [79]	double-blind RCT	Carnosine (800 mg/day in 2 doses) or placebo in addition to risperidone; 10 weeks	70	Carnosine supplementation resulted in a reduction in hyperactivity and non-compliance in children with ASD.
Ghajar et al. (2018) [67]	double-blind RCT	Carnosine (2 g/day in two divided doses) or placebo; 8 weeks	63	Carnosine add-on therapy reduced the primary negative symptoms of patients with schizophrenia.
Ann Abraham et al. (2020) [83]	double-blind RCT	Carnosine (10–15 mg/kg in 2 divided doses/day) or placebo; 2 months	63	No statistically significant difference was observed for any of the outcome measures assessed.
Woo et al. (2015) [69]	two-arm prospective	SSRI; 6 weeks	68/22 (90 total)	A potential was shown to measure therapeutic response. Patients with MDD, after 6 weeks of SSRI treatment, had alterations of amino acids, including beta-alanine (and alanine, beta-aminoisobutyric acid, cystathionine, ethanolamine, glutamic acid, homocystine, methionine, O-phospho-L-serine and sarcosine).
Ali Sisto et al. (2023) [70]	two-arm, prospective	Antidepressant quetiapine; 40 weeks	99/253 (352 total)	The use of any antipsychotic medication was associated with lowered carnosine levels. Elevated serum levels of carnosine were also associated with a longer duration of the depressive episode.

ASD: autism spectrum disorders; MDD: major depressive disorder; RCT: randomized controlled trial; SSRI: selective serotonin reuptake inhibitors.

**Table 4:** Carnosine, beta-alanine and sarcopenia.

Author (Year)	Study Design	Intervention	Number of Patients	Effect
del Favero et al. (2012) [94]	double-blind RCT	Beta-alanine (3.2 g divided into 4 doses/day) or placebo; 12 weeks	18	Supplementation is effective in increasing the muscle carnosine content in healthy elderly subjects, with improvement in exercise capacity.
McCormack et al. (2013) [95]	double-blind RCT, three-arm	(1) ONS (containing 8 oz; 230 kcal; 12 g protein, 31 g cholesterol, 6g fat); 12 weeks (2) ONS plus beta-alanine (800 mg, 2×/day); 12 weeks (3) ONS plus beta-alanine (1200 mg, 2×/day); 12 weeks	60	ONS fortified with beta-alanine may improve physical working capacity, muscle quality and function in older men and women.
Furst et al. (2018) [96]	double-blind RCT	Beta-alanine (2.4 g/day) or placebo; 28 days	12	Supplementation increased exercise capacity and eliminated endurance exercise-induced declines in executive function seen after recovery.

ONS: oral nutritional supplement; RCT: randomized controlled trial.

**Table 5:** The effects of carnosine on oral mucositis, taste disorders, gastritis and gastrointestinal dysfunctions.

Author (Year)	Study Design	Intervention	Number of Patients	Effect
Doi et al. (2015) [108]	two-arm, prospective	1 min ZnC mouth rinse (37.5 mg/10 mL, 4×/day)	32	Grade 3 mucositis was observed less frequently according to clinical findings and symptomatology. ZnC promoted recovery.
Watanabe et al. (2010) [113]	RCT	ZnC oral rinse	16/15 (31 total)	Use of analgesics was less frequent and the amount of food intake was significantly higher. Tumor response rate was not affected in patients receiving ZnC.
Hayashi et al. (2016) [110]	prospective, three-arm	ZnC lozenge (18.75 mg 4×/day), ZnC suspension (75 mg in 4 doses/day)	19/31/16 (66 total)	ZnC lozenge was highly effective for prevention of moderate to severe oral mucositis in patients receiving high-dose chemotherapy for HSCT. The efficacy of lozenge preparation was comparable suspension.
Hayashi et al. (2014) [109]	retrospective	ZnC (500 mg) in 20mL P-AG, mouth rinse	36	Reduced the incidence of moderate-to-severe oral mucositis, and pain was significantly relieved. Incidence of xerostomia and taste disturbance tended to be lowered, but not significantly.
Yanase et al. (2015) [114]	retrospective	60 mL P-AG and 150 mg ZnC (3×/day)	19/19 (38 total)	ZnC highly effective in suppressing the development of radiation esophagitis without affecting the tumor response rate.
Sakagami et al. (2009) [106]	double-blind RCT, multi-center	ZnC (17 mg, 34 mg or 68 mg/day; 12 weeks)	28/27/26/28 (109 total)	An amount of 68 mg of ZnC showed a significant improvement in gustatory sensitivity.
Fujii et al. (2018) [107]	retrospective	ZnC (150 mg; 2×/day), until symptom disappearance	80	The administration of 150 mg of ZnC to patients (with pancreatic cancer treated with fluoropyrimidines) with grade 2 dysgeusia significantly shortened its duration.
Mahmood et al. (2007) [112]	RCT	ZnC (37.5 mg; 2×/day) before and after 5 days of indomethacin treatment (50 mg; 3×/day)	10	ZnC, at concentrations likely to be found in the gut lumen, stabilized gut mucosa.
Tan et al. (2017) [115]	RCT, multi-center	ZnC (150 mg/day) combined with triple therapy; ZnC (300 mg/day) combined with triple therapy; triple therapy	113/108/111 (332 total)	Confirmed the effectiveness of the zinc compound in improving *HP* eradication rate.
Takaoka et al. (2010) [116]	two-arm, prospective	150 mg of ZnC orally	12/28 (40 total)	No significant correlation between improvement of VAS pain score and the zinc concentration in the serum after zinc supplementation.
Masayuki et al. (2002) [117]	two-arm, prospective	ZnC and 2% carmellose sodium	19	ZnC was found to have efficacy and safety as a preventive drug for radiation-induced stomatitis.
Suzuki et al. (2016) [111]	retrospective	P-AG oral rinse	104	P-AG was found to be effective in preventing severe oral mucositis and reducing the irradiation period and median time to discharge after completion of radiotherapy.
Baraniuk et al. (2013) [57]	RCT	Carnosine (500 mg, 1000 mg and 1500 mg increasing at 4-week intervals)	25	Decrease in diarrhea associated with irritable bowel syndrome.
Kitagawa et al. (2021) [118]	RCT, multi-center	ZnC lozenge (18.75 mg) 4×/day, until 35 days after transplantation	47/41 (88 total)	In patients receiving high-dose chemotherapy followed by hematopoietic stem cell transplantation, grade ≥2 oral mucositis was significantly reduced in the intervention group.
Jung et al. (2021) [119]	RCT	ZnC (150 mg/day), pantoprazole or rebamipide (300 mg/day), and pantoprazole	200	ZnC plus PPI treatment showed noninferiority to rebamipide, with PPI treatment of the ulcer healing rate at 4 weeks after endoscopic submucosal dissection.

*HP*: *Helicobacter pylori*; HSCT: hemopoetic stem cell transplantation; P-AG: polaprezinc suspension sodium alginate solution; PPI: proton pump inhibitor; RCT: randomized controlled trial; VAS: visual analogue scale; ZnC: zinc carnosine.

## Data Availability

Not applicable.

## Supplementary Materials

The following supporting information can be downloaded at: https://www.mdpi.com/article/10.3390/nu15071770/s1, Table S1. Molecular functions of carnosine.
