# Supplementary material for: Carnosine and Beta-Alanine Supplementation in Human Medicine: Narrative Review and Critical Assessment
Source: Nutrients. 2023 Apr 5;15(7):1770. doi: 10.3390/nu15071770 (PMC10096773; doi:10.3390/nu15071770)
Supplement: Supplementary file 1 [file nutrients-15-01770-s001.zip › Supplementary_Table S1.pdf]

Table S1. Molecular functions of carnosine.

Author	Year	Ref.	Function
Nagai et al.	1986	[14]	Imunomodulating properties.
Hipkiss et al.	1998	[5]	Inhibits nonenzymic glycosylation and cross-linking of proteins induced by reactive aldehydes, protection against amyloid-induced toxicity.
Holliday et al.	2000	[12]	Prolong cells lifespan and reduce the normal features of senescence.
Nicoletti et al.	2007	[8]	Direct NO-trapping ability.
Corona et al.	2011	[9]	Metal chelating properties (copper and zinc ions), reacts with RONS.
Boldyrev et al.	2013	[10]	pH buffer in skeletal muscle. Regulation of excitation-contraction coupling through calcium ions.
Villari et al.	2014	[13]	Protection against structural protein damage in stress conditions (chemically or thermally induced)
Hipkiss et al.	2016	[15]	Metabolic regulatory activities (glycolytic enzymes, redox biology, glyoxalase activity), protein glycation, apoptosis, gene expression and metastasis. Rejuvenation of senescent cells, telomere shortening, antioxidant activity, carbonyl scavenging, metabolic regulatory activities (glycolysis, mitochondrial activity, ATP), mTOR inhibition.
Abdelkader et al.	2016	[7]	Antiglycation properties, reacts with ALE.
Mikuła-Pietrasik et al.	2018	[18]	Prevention of cell adhesion, migration, invasion and proliferation of senescent ovarian carcinoma cells.
Dolan et al.	2018	[6]	pH regulation, calcium ion regulation, protection against AGE.
Haus et al.	2018	[4]	Reacts with RONS and biomolecule oxidation products. Lowering lipid peroxidation, metabolic and inflammatory outcomes in vivo.
Hwang et al.	2019	[21]	Anti-cancer actions – angiogenesis suppression of bladder cancer cells.
Wu et al.	2019	[19]	Inhibits metastatic cell adhesion and extravasation (human colorectal cancer cells)
Hwang et al.	2019	[21]	Anti-cancer actions – Suppression of angiogenesis.
Li et al.	2020	[17]	Boost the immune surveillance ability of macrophages in cell senescence and clearance.
Turner et al.	2021	[16]	Anti-cancer actions – regulation of energy metabolism, gene expression, proteolysis, clearance of senescent cells.
Aldini et al.	2021	[11]	Anti-inflammatory, antioxidant (through the Nrf2 pathaway), antiglycation and anti-carbonyl effects, pH regulation.
Prakash et al	2021	[20]	Inhibits breast, ovarian, colon, and leukemic cancer cell proliferation; upregulates expression of pro-inflammatory molecules; modulates cytokine secretion.

AGE: advanced glycation end products; ALE: advanced lipoxidation end products; ATP: adenosine triphosphate; NO: nitric oxide; RONS: reactive oxygen and nitrogen species; ROS: reactive oxygen species; mTOR: mammalian target of rapamycin
